# Supplementary material for: Molecular analysis of the ribosome recycling factor ABCE1 bound to the 30S post‐splitting complex
Source: EMBO J. 2020 Feb 17;39(9):e103788. doi: 10.15252/embj.2019103788 (PMC7196836; doi:10.15252/embj.2019103788)
Supplement: Supplementary file 1 — Appendix [file EMBJ-39-e103788-s001.pdf]

Appendix

Table of content

Appendix Figure S1 - Secondary structure of *T. celer* 16S rRNA

Appendix Figure S2 - Sequence alignment of ABCE1 from different species

Appendix Figure S3 - Quality control of *S. solfataricus* ABCE1 variants

Appendix Figure S4 - Structural alignment of ABCE1 with bacterial ABC-importers

Appendix References

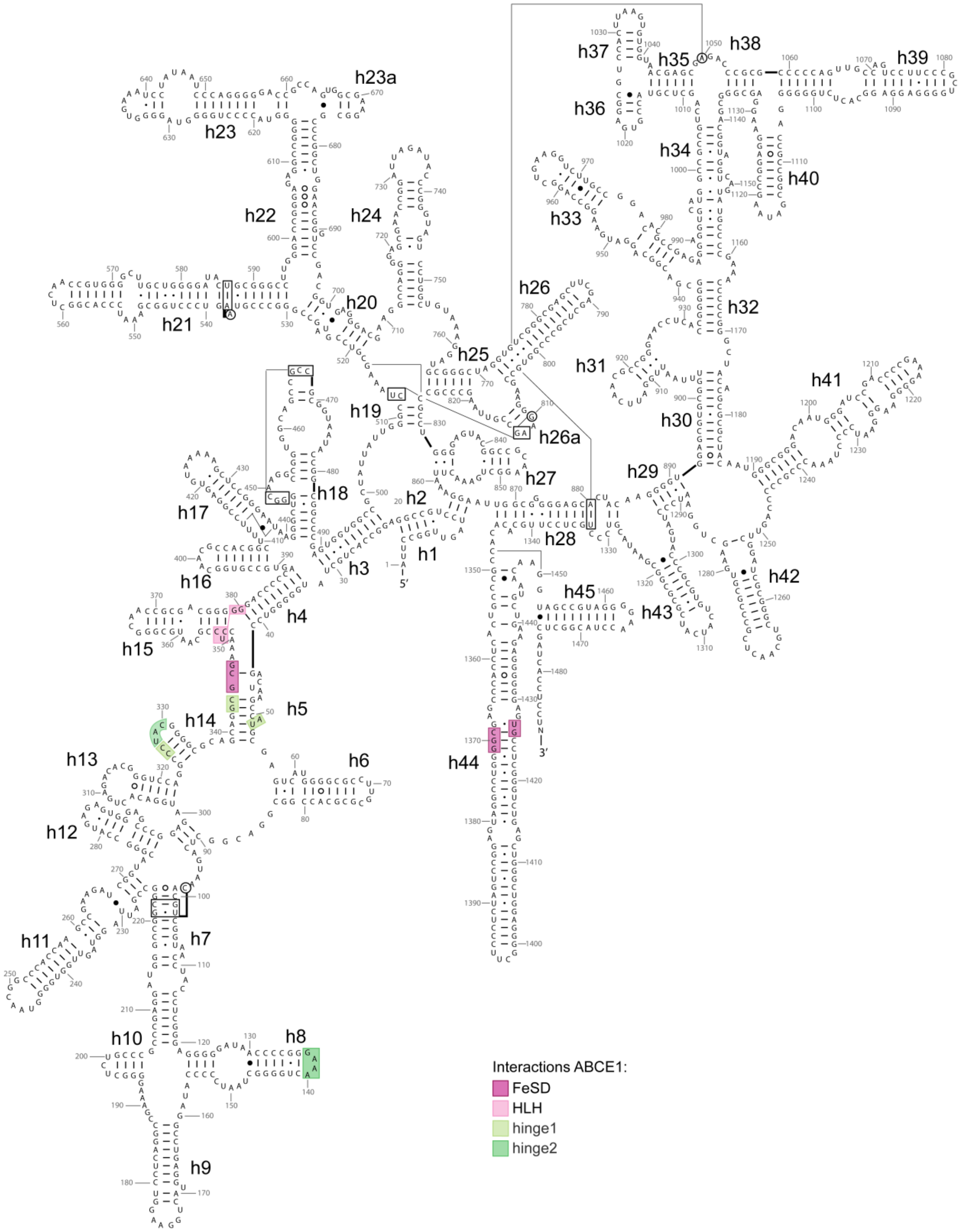

Appendix Figure S1. Secondary structure of *T. celer* 16S rRNA. 1487 nucleic acid residues form the *T. celer* 16S rRNA (Cannone *et al*, 2002). ABCE1-domain interactions with the 16S rRNA are colored according to the domain architecture of ABCE1 in Figure 1. FeSD contacts G345, C346 and G347 of h5, and C1369, G1370, G1371, G1426 and U1427 of h44. HLH motif binds to C352, U253, C354 and G379 of h15, and G380 of h4. Hinge 1 contacts h5 at A51, U52, G343 and C344, and h14 at C326 and C327. Hinge 2 anchors to the ribosome at G137, A138, A139 and A140 of h8, and U328, A329 and C330 of h14.

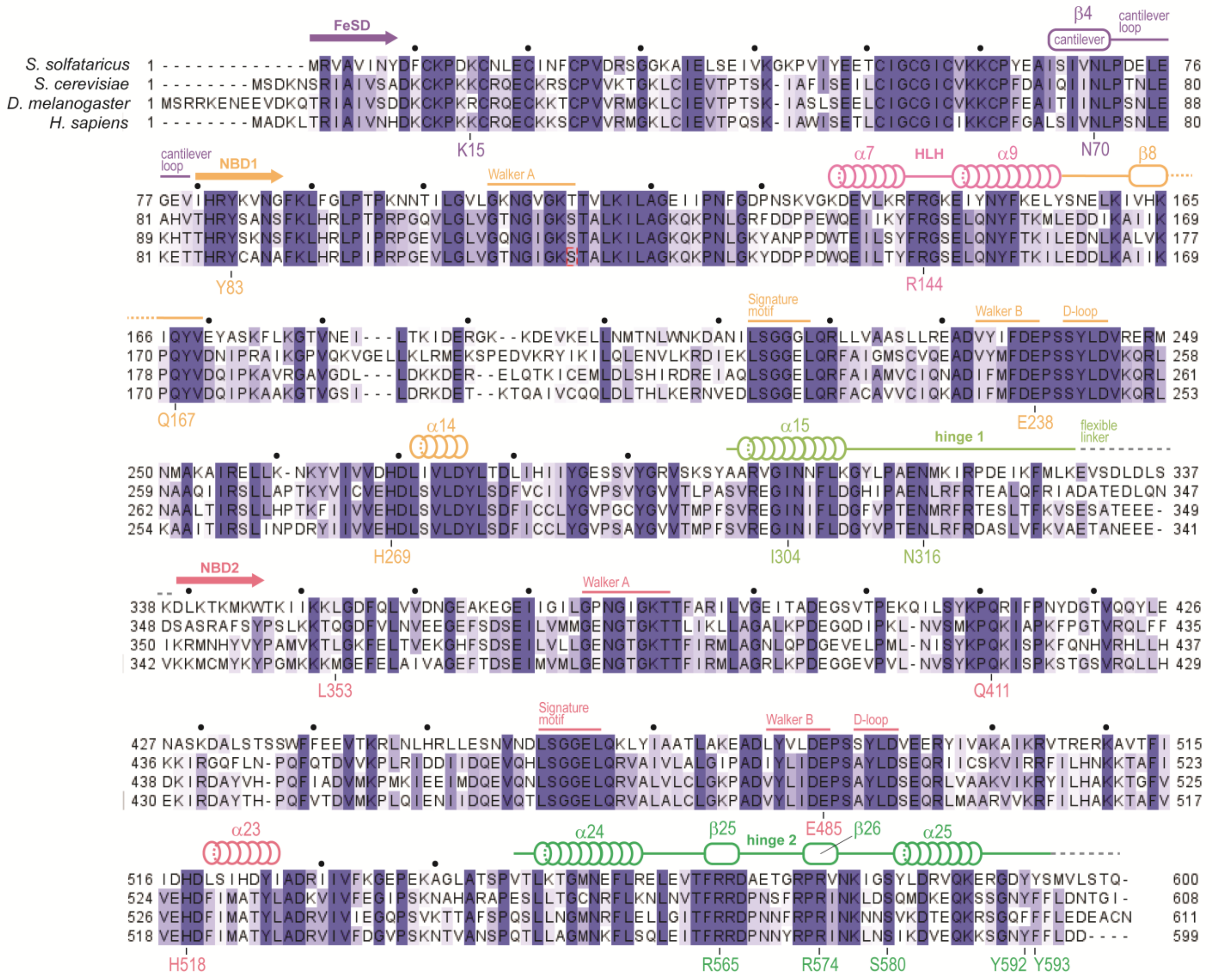

Appendix Figure S2. Sequence alignment of ABCE1 from different species. *S. solfataricus*, *S. cerevisiae*, *D. melanogaster* and *H. sapiens* ABCE1 display strong sequence conservation, illustrated by the shades of blue. Numbering according to *S.s.* ABCE1. Domains are indicated by arrows. Loops are represented by lines, α-helices by tubes and β-sheets by boxes. Conserved motifs, important secondary structure elements (numbered according to (Karcher *et al*, 2008) and colored according to Figure 1) and residues are indicated.

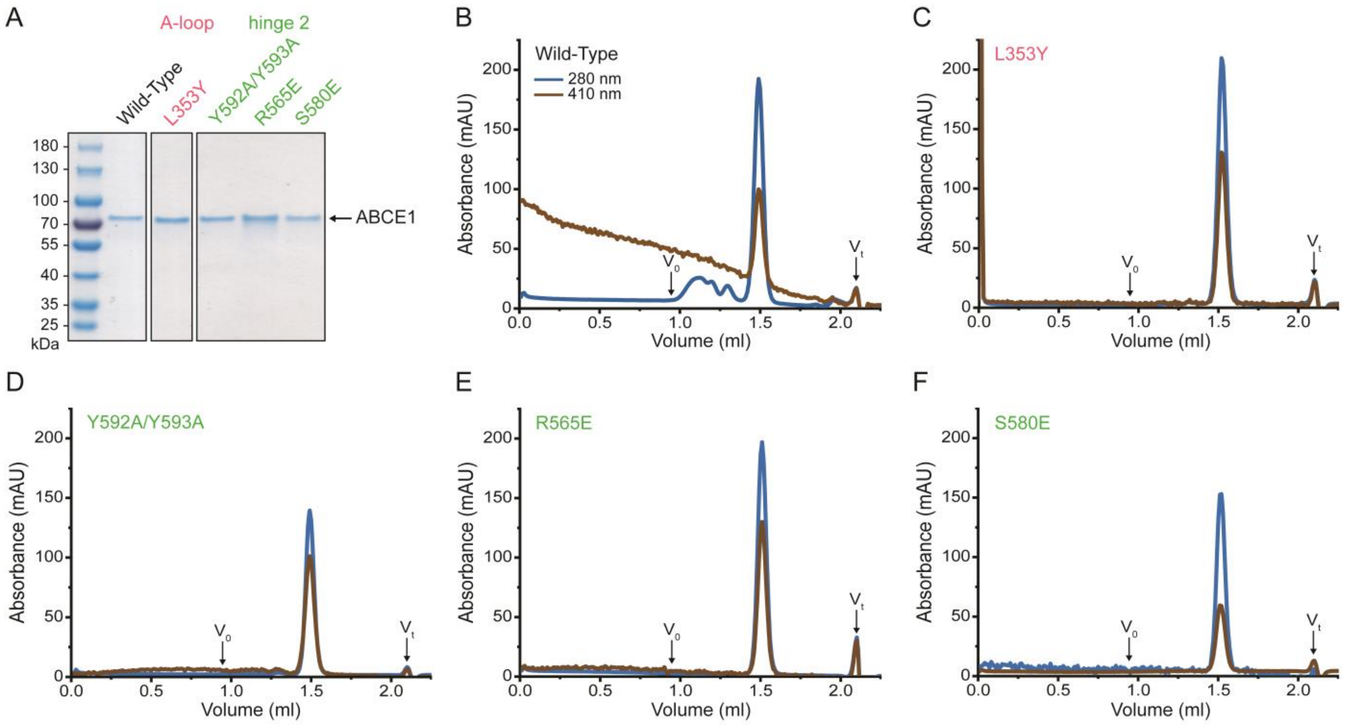

Appendix Figure S3: Quality control of *S. solfataricus* ABCE1 variants. A, Quality of purified ABCE1 variants is assured by single protein bands at the expected molecular weight in SDS-PAGE. **B-F**, All ABCE1 variants elute in single symmetric peaks in size-exclusion-chromatography confirming monodisperse protein samples. Absorbance at 410 nm attests correct assembly of the iron-sulfur clusters.

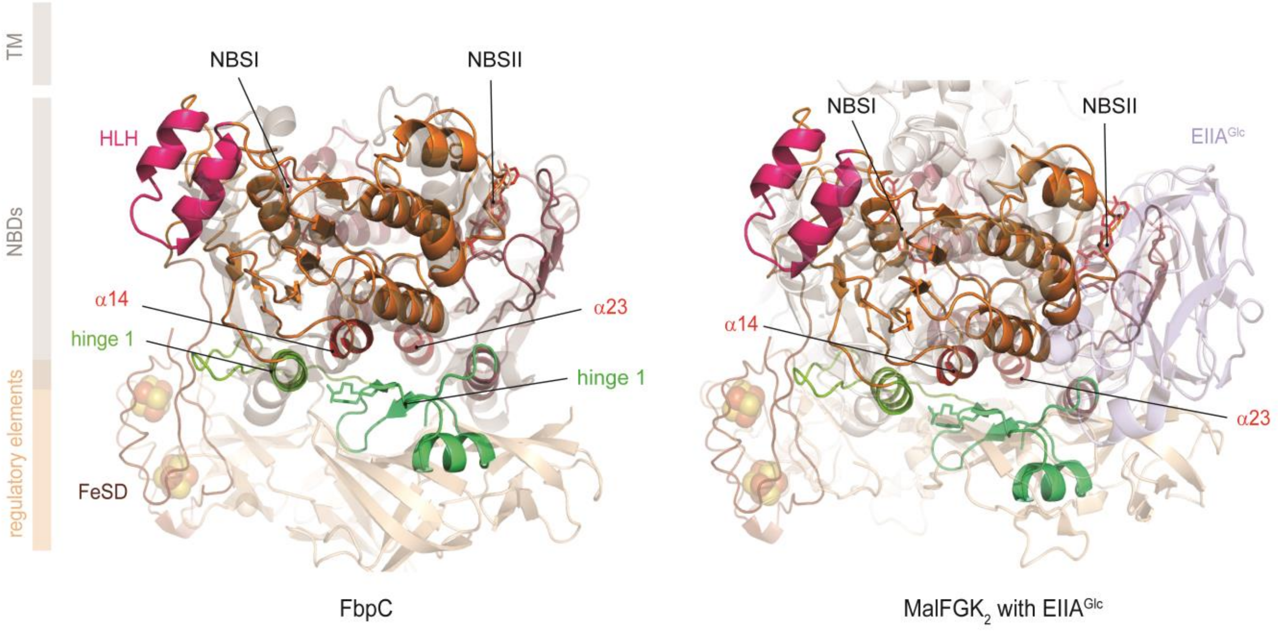

Appendix Figure S4. Structural alignment of ABCE1 with bacterial ABC-importers. Superposition of the NBDs from ABCE1, the iron uptake transporter FbpC (left) of *Neisseria gonorrhoeae* (3FVQ) (Newstead *et al*, 2009), and the maltose transporter $MalFGK_2$ (right) in complex with the glucose-specific phosphotransferase enzyme $EIIA^{Glc}$ from *E. coli* (4JBW) (Chen *et al*, 2013). The hinge regions of ABCE1 are located at the same position as the regulatory elements of the ABC-importers. Thus, hinge 1 and hinge 2 may fulfill regulatory functions in ribosome sensing and communication to the NBSs via α14 and α23, in accordance with the evolution of the ubiquitous ABC-protein system.

Appendix References

Cannone JJ, Subramanian S, Schnare MN, Collett JR, D'Souza LM, Du Y, Feng B, Lin N, Madabusi LV, Muller KM, Pande N, Shang Z, Yu N, Gutell RR (2002) The comparative RNA web (CRW) site: an online database of comparative sequence and structure information for ribosomal, intron, and other RNAs. *BMC bioinformatics* 3: 2

Chen S, Oldham ML, Davidson AL, Chen J (2013) Carbon catabolite repression of the maltose transporter revealed by X-ray crystallography. *Nature* 499: 364-8

Karcher A, Schele A, Hopfner KP (2008) X-ray structure of the complete ABC enzyme ABCE1 from Pyrococcus abyssi. *J Biol Chem* 283: 7962-71

Newstead S, Fowler PW, Bilton P, Carpenter EP, Sadler PJ, Campopiano DJ, Sansom MS, Iwata S (2009) Insights into how nucleotide-binding domains power ABC transport. *Structure* 17: 1213-22
