# Supplementary material for: Molecular analysis of the ribosome recycling factor ABCE1 bound to the 30S post‐splitting complex
Source: EMBO J. 2020 Feb 17;39(9):e103788. doi: 10.15252/embj.2019103788 (PMC7196836; doi:10.15252/embj.2019103788)
Supplement: Supplementary file 2 — Expanded View Figures PDF [file EMBJ-39-e103788-s002.pdf]

Expanded View Figures

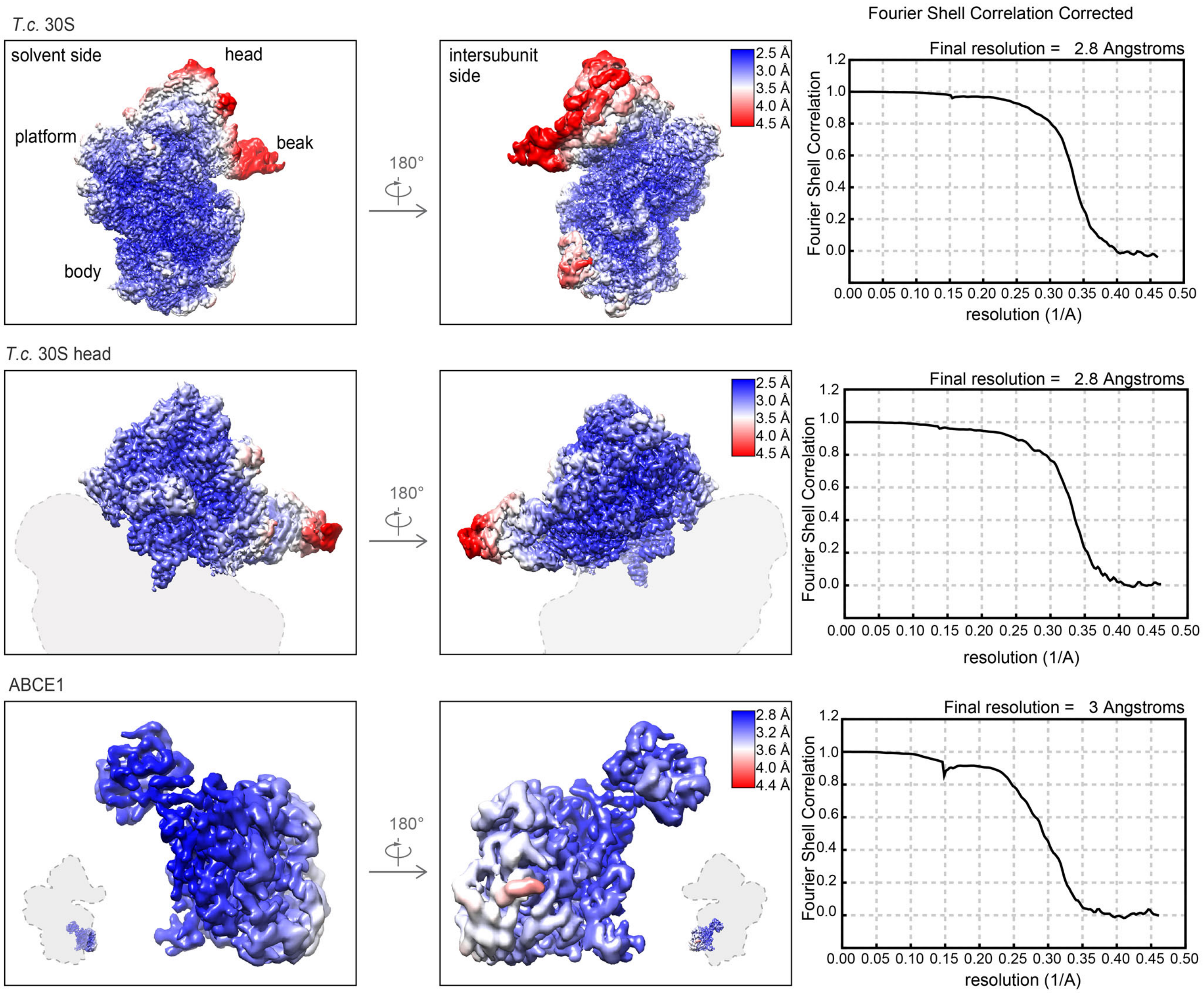

Figure EV1. Local resolution of the post-SC.
Cryo-EM maps of the overall 30S-ABCE1 post-SC (top) and locally refined 30S head (middle) and ABCE1 (bottom) moieties. Maps are colored and filtered according to local resolution, and corresponding gold standard FSC curves are shown. Using focused refinement, local resolution of the 30S head and ABCE1 was improved from approx. 4–6 Å to 3.0 Å and 2.8 Å, respectively.

 © 2020 The Authors

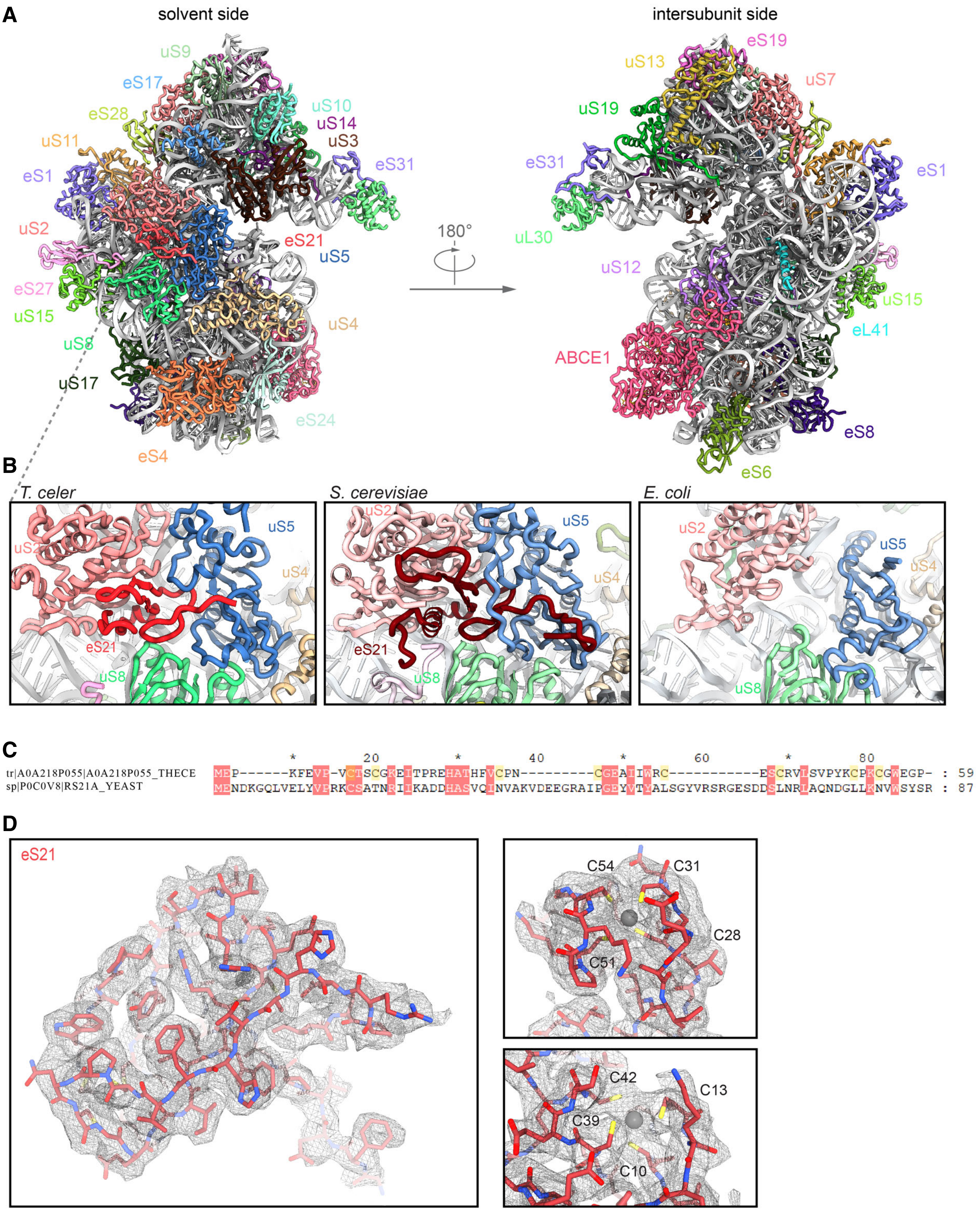

Figure EV2. Location of eS21 and molecular model of the *T. celer* 30S subunit.

A *T. celer* 30S subunit contains 28 ribosomal proteins, including the large subunit protein eL41.

B Close-up view on eS21 located at the solvent side between uS2, uS5, and uS8. Comparison with other species reveals that the respective position at the ribosome is not occupied in *E. coli*, but by eS21 in *S. cerevisiae*.

C Sequence alignment of *T. celer* eS21 and *S. cerevisiae* eS21a shows low homology, indicating that the two proteins are only weakly related.

D Cryo-EM density for e21 and fit of the *de novo* model. The protein forms two zinc-binding pockets, each coordinated by four cysteines.

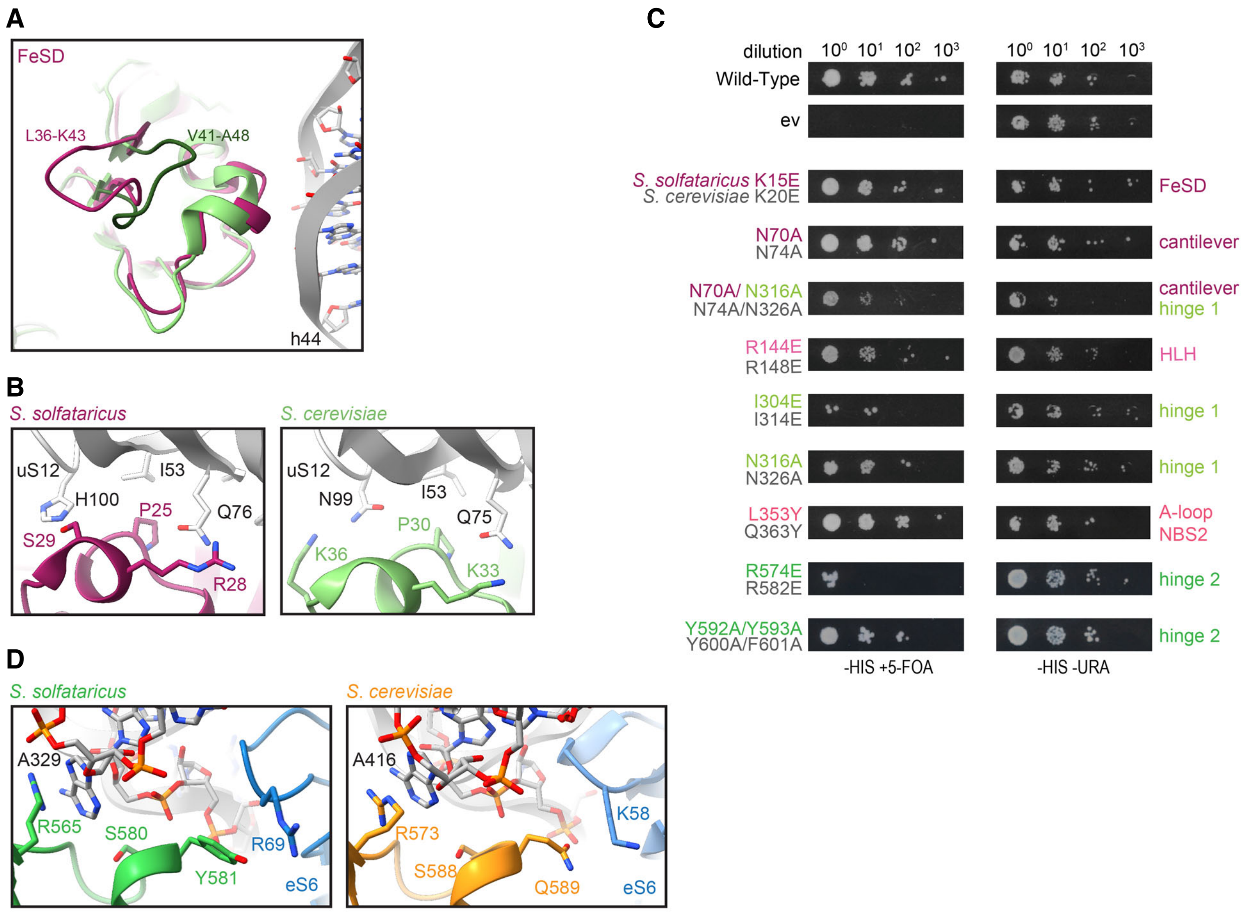

Figure EV3. Conserved interactions of ABCE1 with 30S are essential for ABCE1 function.

A FeSD interactions are conserved between *S. solfataricus* and *S. cerevisiae*, except for the loops L36–K43 (*S. solfataricus*) and V41-A48 (*S.c.*), which face away from the ribosome and vary in sequence and structure.

B The interaction between S29 and H100 (uS12) is substituted by K36 and N99 (uS6) in yeast, indicating co-evolution of ABCE1 and the ribosome.

C Yeast plasmid shuffling assay illustrates cell viability and growth either dependent or independent on the plasmid with mutant ABCE1 in the presence or absence of 5-FOA, respectively.

D Stacking of Y581 with R69 (eS6) occurs in yeast as Q589 with K58 (eS6), giving another hint for ABCE1-ribosome co-evolution in order to maintain essential interactions.

Data Information: In (C), data are representative for a set of two independent experiments.

 © 2020 The Authors

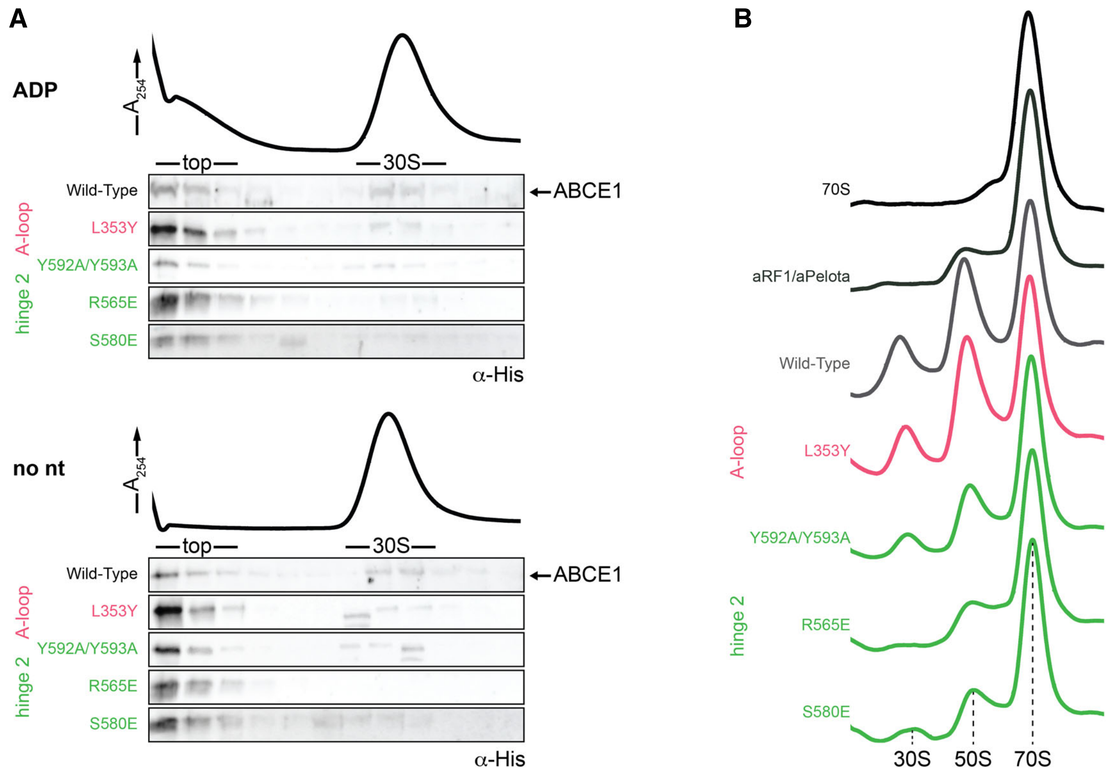

Figure EV4. Detailed *in vitro* biochemical characterization of ABCE1 variants.

A As wild-type ABCE1, all variants are unable to bind 30S ribosomes in the presence of ADP or in the absence of nucleotide (no nt), thereby excluding that the respective mutation does not lead to unspecific binding to the ribosome.

B Examples of sucrose density gradient profiles of 70S splitting reactions illustrate reduced splitting efficiencies of hinge 2 mutants compared to wild-type ABCE1. SDG profile of the background control (aRF1/aPelota) is similar to R565E, highlighting its essential anchoring function (see Fig 3C).

Source data are available online for this figure.

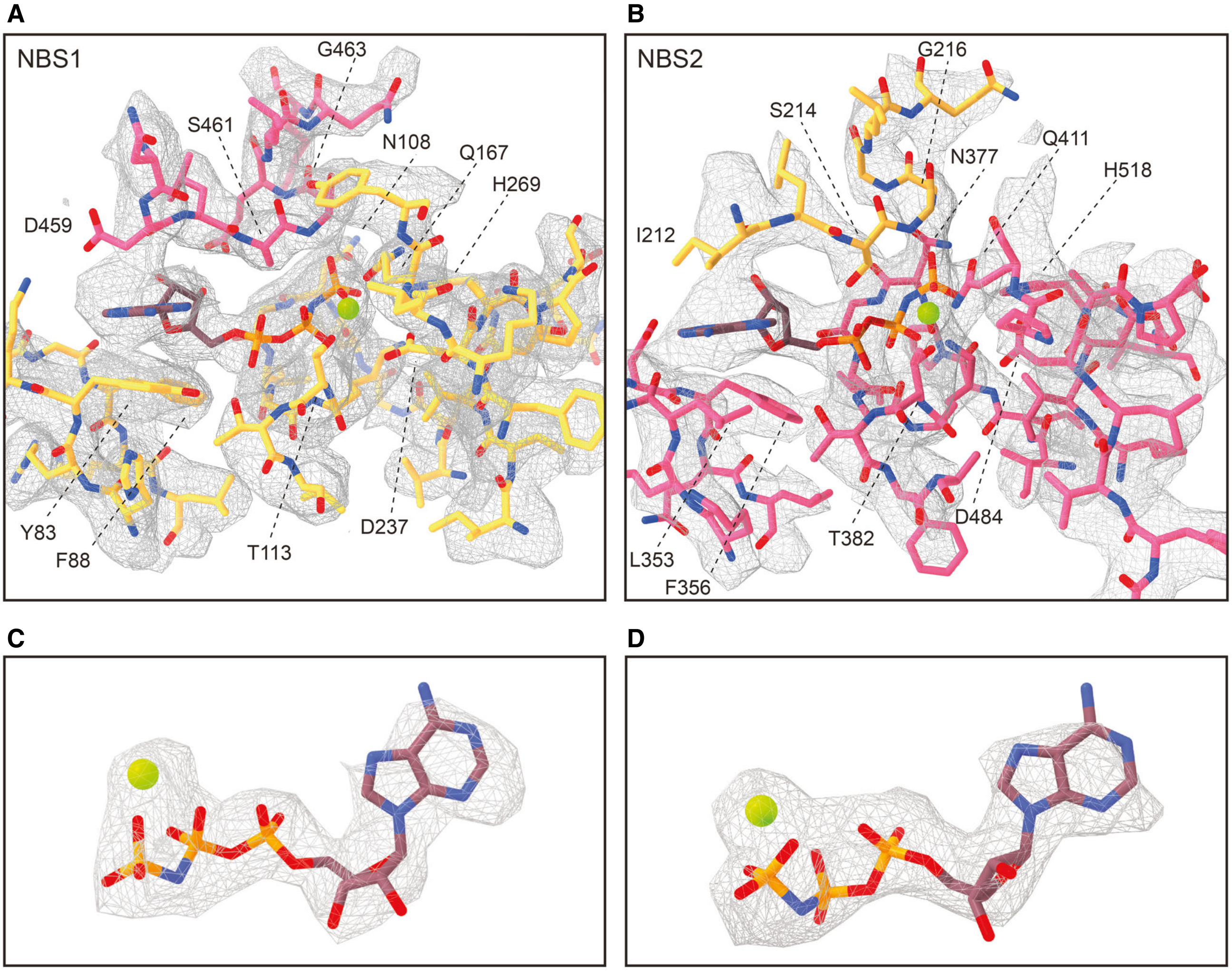

Figure EV5. Fitting of NBSI and NBSII in the cryo-EM density.

A Zoomed view on the model for NBSI fit into the electron density map shown in the same view as in Fig 3I. Residues from NBD1 and NBD2 are shown in gold and punch, respectively, and residues contributing to Mg^{2+}-AMP-PNP binding are labeled.

B Same as in (A) but for NBSII, corresponding to Fig 3J.

C, D Electron density and fit model for isolated Mg^{2+}-AMP-PNP from both NBSI (C) and NBSII (D). We clearly observe density for the Mg^{2+}-ion coordinated by the γ- and β-phosphates of the trinucleotide in both NBSs.

 © 2020 The Authors
