# Supplementary material for: Molecular analysis of the ribosome recycling factor ABCE1 bound to the 30S post‐splitting complex
Source: EMBO J. 2020 Feb 17;39(9):e103788. doi: 10.15252/embj.2019103788 (PMC7196836; doi:10.15252/embj.2019103788)
Supplement: Supplementary file 3 — Movie EV1 [file EMBJ-39-e103788-s003.zip › Movie_EV1.docx]

**Movie EV1 | Model for ribosome splitting by ABCE1.** Conformational changes within ABCE1 during the transition from the pre-splitting complex (3JAH (Brown et al, 2015)) to the post-splitting complex. Before splitting the nucleotide-binding sites (NBSs) are in a semi-closed state. Closure of NBSII triggers an allosteric chain within ABCE1 leading to a tight closure of NBSI and the FeSD is displaced. Simultaneously, the hinge region opens up and rearranges ABCE1 in the ribosomal subunit cleft. Thereby the subunits are split apart and the FeSD is positioned at h44.
