# Supplementary material for: Molecular analysis of the ribosome recycling factor ABCE1 bound to the 30S post‐splitting complex
Source: EMBO J. 2020 Feb 17;39(9):e103788. doi: 10.15252/embj.2019103788 (PMC7196836; doi:10.15252/embj.2019103788)
Supplement: Supplementary file 6 — Source Data for Figure 3 [file EMBJ-39-e103788-s004.zip › Figure_3E_Source_Data.pdf]

Source Data Figure 3

Immunoblots of 30S binding assay with **AMPPNP**.

Sucrose-density gradient fractions (lanes 2-14) were run on 12.5% SDS-PAGEs, blotted, blocked, and incubated with anti-His and anti-rabbit antibodies (see Material and Methods). Luminescence was imaged using a Fusion FX (Vilber). In the first lane PageRuler™ Prestained Protein Ladder, 10 to 180 kDa (26616, Thermo Fisher) is loaded. The last lane is an ABCE1-His_6 control.

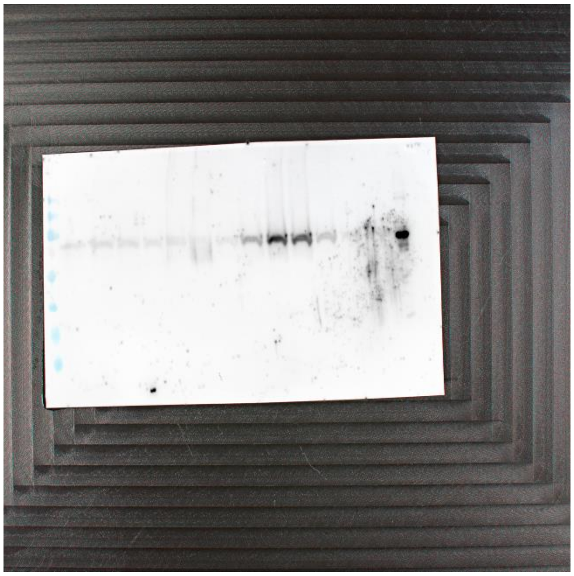

Wild-Type

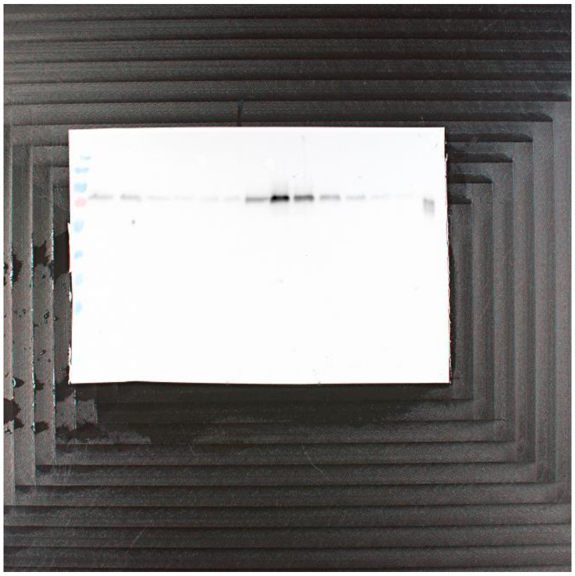

Y592A/Y593A

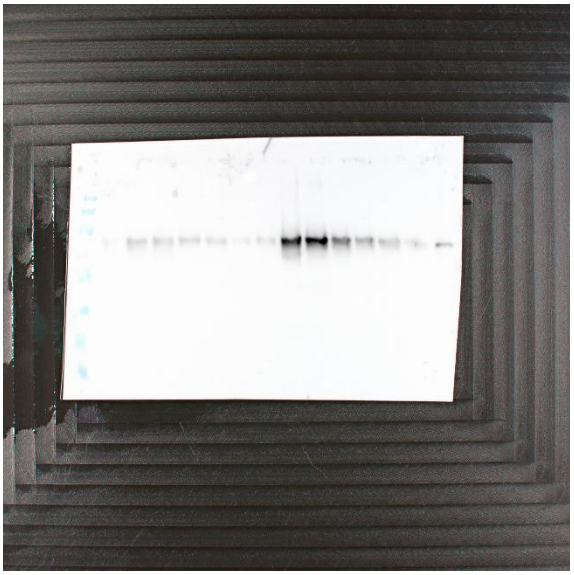

L353Y

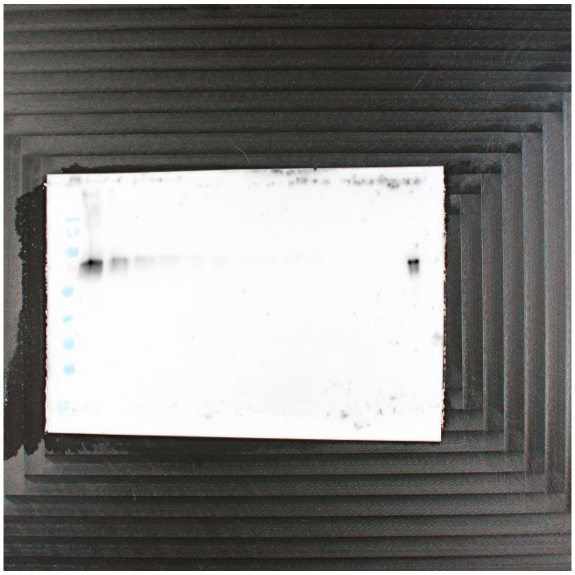

R565E

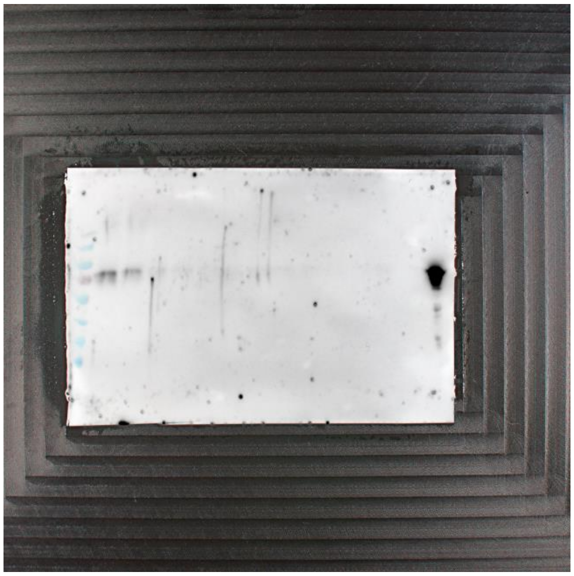

S580E
